# Alagille Syndrome: A Case Report Highlighting Dysmorphic Facies, Chronic Illness, and Depression

**DOI:** 10.1155/2016/1657691

**Published:** 2016-11-27

**Authors:** James J. Bresnahan, Zachary A. Winthrop, Rabia Salman, Salman Majeed

**Affiliations:** ^1^The Pennsylvania State University College of Medicine, Hershey, PA, USA; ^2^Nishtar Medical College, Multan, Pakistan; ^3^Department of Psychiatry, The Pennsylvania State University College of Medicine, Hershey, PA, USA

## Abstract

Alagille syndrome is a rare multisystem disorder affecting the liver, heart, vertebrae, eyes, and face. Alagille syndrome shares multiple phenotypic variants of other congenital or chronic childhood illnesses such as DiGeorge syndrome, Down syndrome, spina bifida, type 1 diabetes mellitus, and cystic fibrosis. All of these chronic illnesses have well-established links to psychiatric conditions. There are few community resources for Alagille patients, as it is an extremely rare condition. Despite the overlap with other chronic childhood illnesses, the psychiatric manifestations of Alagille syndrome have not been previously discussed in literature. The current study is a case report of a twelve-year-old female hospitalized in our pediatric psychiatric hospital for suicidal ideation with intent and plan. The patient had major depressive disorder, anxiety, other specified feeding and eating disorder, and attention-deficit/hyperactive disorder.

## 1. Introduction

Alagille syndrome, also known as arteriohepatic dysplasia, is a rare multisystem disorder with varying degrees of penetrance. The most common symptoms associated with this syndrome are cholestasis (the obstruction or slowing of biliary flow), congenital heart disease (pulmonary artery stenosis), butterfly shaped vertebrae, anterior chamber eye defects, and dysmorphic facies. In most cases, the diagnosis is clinical and almost 90% of cases are due to mutations in JAG1 (20p12) that are inherited in an autosomal dominance pattern [1]. The estimated prevalence is 1:70,000–100:000 [2].

**Figure 1:**
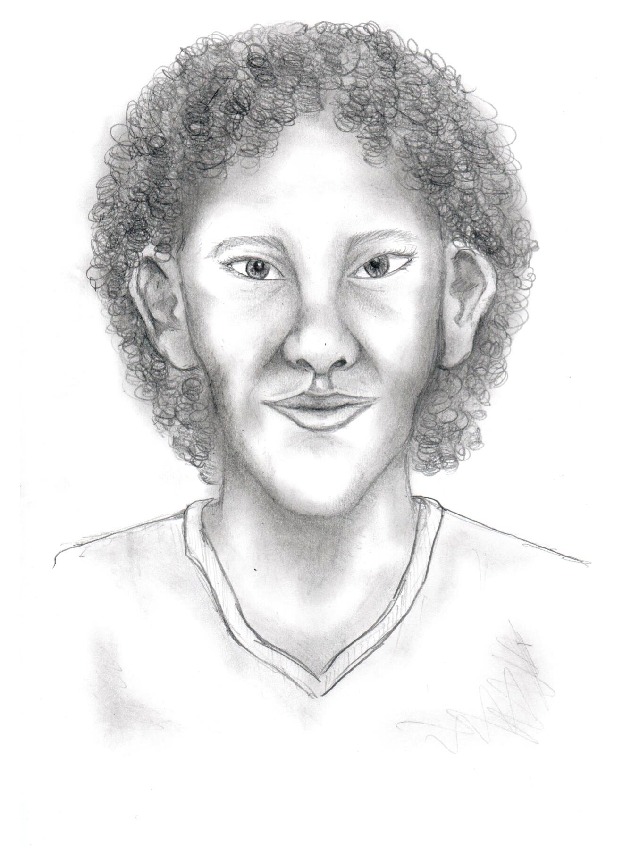
Artist rendition of the facial features associated with Alagille syndrome.

The facial features associated with Alagille syndrome are notable (Table 1, Figure 1). They include a broad forehead, deep-set eyes, upslanting palpebral fissures, prominent ears, bulbous tipped nose, and a pointed chin. Additionally, children with Alagille syndrome have both increased frequency of intellectual disability and increased frequency of motor delay when compared to the general population [3].

Previous studies have shown body image [4–7], physical illness [8–15], chronic medical illness [16–23], and intellectual disability [24, 25] to be independent risk factors for low self-esteem and depression in the pediatric population. Alagille syndrome displays characteristics of childhood chronic medical illness, dysmorphic facies, and potentially cognitive and motor delay in comparison to age-matched peers.

Despite the overlap in features with many other chronic conditions that are well linked to psychiatric conditions, there is no mention of self-esteem, depression, or other psychiatric manifestations of Alagille syndrome in published studies. The current study is a case report of a pediatric Alagille patient who was hospitalized for suicidal ideation (SI) with intent and plan.

## 2. Case Report

### 2.1. History

The patient is a 12-year-old female and was voluntarily admitted to our pediatric psychiatric institute for hospitalization in early 2016 after presenting to an outpatient service with SI, intent, and a plan to overdose on medications and suffocate herself with a plastic bag. This was her first inpatient hospitalization, but she carried the diagnoses of major depressive disorder and anxiety before admission. She was previously followed up by an outside psychiatrist and counselor beginning at age eight for 1-2 sessions per month. She completed a partial hospitalization program one year prior to admission.

The patient revealed that her depressive symptoms primarily revolved around her self-image. The patient had a tumultuous neonatal course that involved multiple cardiac and hepatic issues. After being worked up by our children's cardiology group, the patient was diagnosed with Alagille syndrome in 2005 at the age of 3. In addition to cardiac and hepatic manifestations, Alagille syndrome is associated with dysmorphic facies.

The patient believed her characteristic facial features made her “ugly.” Of the features associated with Alagille syndrome, the patient had a broad forehead, deep-set eyes, upslanting palpebral fissures, prominent ears, bulbous tipped nose, and a pointed chin (Figure 1, Table 1). She also noted that she typically felt isolated and alone since she had facial characteristics that were different from other people. She mentioned that while some other people have diseases that were “only on the inside,” her disease was “visible to everyone.” Additionally, the only other person the patient knew with Alagille syndrome was her mother. Her mother had less manifestations of the syndrome and was not diagnosed until she was an adult. Since her facial characteristics were more pronounced, she felt like “there was nothing she could do that would make her feel normal” and that “she would never be pretty.”

Furthermore, the patient confided that having multiple physicians visits each year contributed to her anxiety and depression. She had a regular follow-up with a pediatric cardiologist, pediatric hepatologist, and pediatric ophthalmologist in addition to her primary care and acute visits with her pediatrician. In all, the patient had well over 100 office visits in twelve years. She also has a notable surgical history including bilateral pulmonary artery branch stents, which were both replaced in the last two years.

The patient endorsed being bullied by people at her school as well as being cyber-bullied. Like most teenagers, our patient spent a significant amount of time on social medial. She printed out derogatory comments that other people would leave on her pictures when they pertained to her looks and often read through them, inducing depressive thoughts. While planning her suicide she made a video proclaiming her death and posted it to various social media accounts.

On admission, the patient reported anhedonia, feelings of guilt and worthlessness, poor energy, and passive SI for at least one month with intermittent SI for the last two months. She engaged in self-injurious behavior over the last year by cutting her bilateral wrists and upper legs.

The patient denied homicidal ideation, manic or hypomanic episodes, compulsive habits, posttraumatic stress symptoms, or recent panic attacks. The patient did endorse self-inducing vomiting and self-restriction of caloric intake secondary to believing she is overweight. Her parents were divorced and disharmonious. Additionally, she experienced verbal abuse from her father, witnessed the effects of drug abuse in her household as a child, and was of low socioeconomic status (SES). Initial labs were done to rule out underlying medical causes for depression, including hypothyroidism (TSH), metabolic deficiencies (B12, Folate, Fe, Na, Hgb, and Hct), drug toxicity (urine drug screen), and pregnancy (beta-HCG).

### 2.2. Mental Status Exam

On exam, the patient had poor eye contact and maintained a downcast gaze throughout the interview. She spoke fluently with decreased tone, decreased rate, and flat prosody. The majority of her answers were mumbled. She spoke only to the questions asked and did not elaborate. She endorsed a depressed mood, which has been about the same for the last month. Her affect was restricted and congruent with her mood. Her cognition was grossly intact. She appeared to have decreased concentration. Her impulse control was fair but insight and judgment were both poor. She denied psychotic symptoms and showed no apparent symptoms of psychosis. An intelligence quotient (IQ) test was performed as part of an outpatient work-up. The results of her IQ test were not readily available, although her mother reported she scored poorly and felt she was not at the same intellectual level as her peers. The patient reported poor grades in school (C-/D student), which her mother confirmed.

## 3. Discussion

During the preteen and teenage years, adolescents often have a higher sensitivity to criticism by peers and are more critical of themselves. When a dysmorphic appearance compounds normal teenage physiology, there is the potential for increased sensitivity to criticism and self-judgement. The patient in the current study developed an altered self-concept, which then deflated her self-esteem and confidence leading to multiple psychiatric illnesses.

It has been previously established that people with physical injuries that affect body image such as amputees [4], spinal cord injury [5], traumatic brain injury [15], osteoarthritis [9], and low back pain [10] view their body image differently after the event or procedure that produced the limitation and have lower self-esteem.

Prior studies have commented on facial features in regard to physical appearance and social interactions. Gavric et al. found physical appearance to be an important factor in establishing social interactions for adolescents and young adults. Specifically, they found dental esthetics, dental self-confidence, and facial type to be significant contributors to self-esteem [7]. Furthermore, female adolescents from various countries find their faces to be most closely correlated with overall body dissatisfaction [26].

Approximately 1% of all live births exhibit a minor or major congenital anomaly and approximately one-third display craniofacial abnormalities. In total, there are more than 700 distinct craniofacial syndromes [27]. Dysmorphic facies in more common congenital disorders like DiGeorge syndrome, or velocardiofacial syndrome (VCFS), and Down syndrome, or trisomy 21, have drawn interest from psychiatric researchers. Adolescents with VCFS are now recognized as being at an increased risk for depression [28, 29], anxiety [28, 29], and ADHD [29], though the most distinctive feature among this population is psychosis [28, 29]. Alagille, VCFS, and trisomy 21 all have varying degrees of intellectual disability. Adolescents with trisomy 21 [24, 30, 31] and other forms of intellectual disability [25] are at an increased risk for developing depression. Communication barriers provide a unique challenge in the diagnosis and treatment of these patients.

Chronic illness in childhood is also an independent risk factor for depression [32]. Common chronic illnesses of childhood include cystic fibrosis [23], diabetes mellitus type 1 [19, 20], cerebral palsy [33], and spina bifida [34], all of which have been linked to altered self-concept, decreased self-esteem, and depression in adolescents. Self-concept, which is the descriptive aspect of one's self, and self-esteem, which is the personal evaluative perspective of self-concept, are both closely related to depression in adolescents [35–38]. This correlation holds true across countries [39, 40], pubertal status, and when adjusted for relevant variables such as social support, maternal depression, stressful events, and relational victimization [39].

Adolescents with chronic illnesses necessitate a particular degree of resilience not often seen in their age-matched peers. Resilience is the process of positive adjustment in the context of significant life adversity and is an important element of positive mental health. Resilience has been relatively underresearched among individuals with congenital conditions but has been shown to increase self-esteem and protect against depression in high risk patients [34]. Adolescents with high reported self-concept also report high life satisfaction [41]. Increased self-esteem [40] and exercise [42] have both been shown to ward off depressive symptoms. In patients like the one in the current study, encouraging habits that increase self-concept and self-esteem, such as forming relationships and exercising, appear to be reasonable suggestions that complement the standard of care.

The patient in the current study suggested that she often felt alone secondary to the rarity of her chronic illness. There is a national organization, the Alagille Syndrome Alliance (ALGSA), which was founded in 1993. ALGSA began holding a national symposium in 1999, which consisted of just 27 attendees, including the ALGSA board of directors. Symposia run only every couple of years but have grown to now include a camp for children with Alagille [43]. ALGSA also has an online registry for current research studies [44]. ALGSA provides a unique opportunity to connect with others around the country who work through many of the same issues regarding the chronicity of the illness and identifiable facial features. Our patient did not know of this organization. Providers for other chronic illnesses often suggest attending camps and local meetings for patients in their pediatric population to aid with social integration and self-actualization.

Despite the similarities to physical ailments, dysmorphic facial syndromes, and those with chronic illness, psychiatric diseases have never been described in patients with Alagille syndrome. The established literature suggests that multiple factors would predispose an Alagille patient to developing psychiatric illness. In this case, a combination of low self-esteem secondary to dysmorphic facies and the burden of chronic illness, as well as parental disharmony and low SES, likely coalesced to manifest as major depressive disorder, anxiety, ADHD, and potentially other specified feeding and eating disorder.

Alagille syndrome, like many other congenital disorders, may be best treated through a multidisciplinary approach with the goal of societal reintegration. The physicians who care for Alagille patients should be aware of the psychiatric potential of the syndrome given the multiple areas of overlap with other established psychiatric risk factors and monitor them appropriately. ALGSA is a useful resource for patients with Alagille syndrome. In addition to following up closely with an outpatient psychiatrist, we suggested our patient would benefit from face-to-face meetings with other Alagille patients and others with rare congenital disorders in hopes of forming therapeutic alliances. By meeting other adolescents with similar facial features and life circumstances, we hope the patient in the current study will continue to improve her psychiatric health.

## Figures and Tables

**Table 1:** Dysmorphic facial features associated with Alagille syndrome.

	Description
Forehead	Broad
Ears	Prominent, large
Nose	Bulbous tipped
Chin	Pointed, which gives the face a triangular appearance
Eyes	Deep-set, palpebral fissures, sometimes upslanting; anterior chamber defects with posterior embryotoxin

## References

[1] Turnpenny P. D., Ellard S. (2012). Alagille syndrome: pathogenesis, diagnosis and management. *European Journal of Human Genetics*.

[2] Danks D. M., Campbell P. E., Jack I., Rogers J., Smith A. L. (1977). Studies of the aetiology of neonatal hepatitis and biliary atresia. *Archives of Disease in Childhood*.

[3] Alagille D., Estrada A., Hadchouel M., Gautler M., Odièvre M., Dommergues J. P. (1987). Syndromic paucity of interlobular bile ducts (Alagille syndrome or arteriohepatic dysplasia): review of 80 cases. *The Journal of Pediatrics*.

[4] Holzer L. A., Sevelda F., Fraberger G., Bluder O., Kickinger W., Holzer G. (2014). Body image and self-esteem in lower-limb amputees. *PLoS ONE*.

[5] Huang C.-Y., Chen W.-K., Lu C.-Y. (2015). Mediating effects of social support and self-concept on depressive symptoms in adults with spinal cord injury. *Spinal Cord*.

[6] Farrar S., Stopa L., Turner H. (2015). Self-imagery in individuals with high body dissatisfaction: the effect of positive and negative self-imagery on aspects of the self-concept. *Journal of Behavior Therapy and Experimental Psychiatry*.

[7] Gavric A., Mirceta D., Jakobovic M., Pavlic A., Zrinski M. T., Spalj S. (2015). Craniodentofacial characteristics, dental esthetics-related quality of life, and self-esteem. *American Journal of Orthodontics and Dentofacial Orthopedics*.

[8] Carroll P., Tiggemann M., Wade T. (1999). The role of body dissatisfaction and bingeing in the self-esteem of women with type II diabetes. *Journal of Behavioral Medicine*.

[9] Carr A. J. (1999). Beyond disability: measuring the social and personal consequences of osteoarthritis. *Osteoarthritis and Cartilage*.

[10] Schiphorst Preuper H. R., Reneman M. F., Boonstra A. M. (2008). Relationship between psychological factors and performance-based and self-reported disability in chronic low back pain. *European Spine Journal*.

[11] Ferro M. A., Boyle M. H. (2015). The impact of chronic physical illness, maternal depressive symptoms, family functioning, and self-esteem on symptoms of anxiety and depression in children. *Journal of Abnormal Child Psychology*.

[12] Pinquart M., Shen Y. (2011). Depressive symptoms in children and adolescents with chronic physical illness: an updated meta-analysis. *Journal of Pediatric Psychology*.

[13] Hysing M., Elgen I., Gillberg C., Lie S. A., Lundervold A. J. (2007). Chronic physical illness and mental health in children. Results from a large-scale population study. *Journal of Child Psychology and Psychiatry and Allied Disciplines*.

[14] Hayter M. R., Dorstyn D. S. (2014). Resilience, self-esteem and self-compassion in adults with spina bifida. *Spinal Cord*.

[15] Ponsford J., Kelly A., Couchman G. (2014). Self-concept and self-esteem after acquired brain injury: a control group comparison. *Brain Injury*.

[16] Pérez-Marín M., Gómez-Rico I., Montoya-Castilla I. (2015). Type 1 diabetes mellitus: psychosocial factors and adjustment of the pediatric patient and his/her family. Review. *Archivos Argentinos de Pediatria*.

[17] Rassart J., Luyckx K., Moons P., Weets I. (2014). Personality and self-esteem in emerging adults with Type 1 diabetes. *Journal of Psychosomatic Research*.

[18] Bode C., van der Heij A., Taal E., van de Laar M. A. F. J. (2010). Body-self unity and self-esteem in patients with rheumatic diseases. *Psychology, Health and Medicine*.

[19] Lernmark B., Persson B., Fisher L., Rydelius P.-A. (1999). Symptoms of depression are important to psychological adaptation and metabolic control in children with diabetes mellitus. *Diabetic Medicine*.

[20] Schiffrin A. (2001). Psychosocial issues in pediatric diabetes. *Current Diabetes Reports*.

[21] Lukoo R. N., Ngiyulu R. M., Mananga G. L. (2015). Depression in children suffering from sickle cell anemia. *Journal of Pediatric Hematology/Oncology*.

[22] Rao C., Ramu S. A., Maiya P. P. (2011). Depression in adolescents with chronic medical illness. *International Journal of Adolescent Medicine and Health*.

[23] Duff A. J. A., Abbott J., Cowperthwaite C., Sumner C., Hurley M. A., Quittner A. (2014). Depression and anxiety in adolescents and adults with cystic fibrosis in the UK: a cross-sectional study. *Journal of Cystic Fibrosis*.

[24] Cooper S.-A., Collacott R. A. (1994). Clinical features and diagnostic criteria of depression in Down's syndrome. *British Journal of Psychiatry*.

[25] Sovner R., Hurley A. D. (1983). Do the mentally retarded suffer from affective illness?. *Archives of General Psychiatry*.

[26] Mellor D., Waterhouse M., Mamat N. H. B. (2013). Which body features are associated with female adolescents' body dissatisfaction? A cross-cultural study in Australia, China and Malaysia. *Body Image*.

[27] Trainor P. A., Andrews B. T. (2013). Facial dysostoses: etiology, pathogenesis and management. *American Journal of Medical Genetics, Part C: Seminars in Medical Genetics*.

[28] Fabbro A., Rizzi E., Schneider M., Debbane M., Eliez S. (2012). Depression and anxiety disorders in children and adolescents with velo-cardio-facial syndrome (VCFS). *European Child and Adolescent Psychiatry*.

[29] Antshel K. M., Fremont W., Roizen N. J. (2006). ADHD, major depressive disorder, and simple phobias are prevalent psychiatric conditions in youth with velocardiofacial syndrome. *Journal of the American Academy of Child and Adolescent Psychiatry*.

[30] Walker J. C., Dosen A., Buitelaar J. K., Janzing J. G. E. (2011). Depression in Down syndrome: a review of the literature. *Research in Developmental Disabilities*.

[31] Collacott R. A., Cooper S.-A. (1992). Adaptive behavior after depressive illness in Down's syndrome. *Journal of Nervous and Mental Disease*.

[32] Ferro M. A., Gorter J. W., Boyle M. H. (2015). Trajectories of depressive symptoms during the transition to young adulthood: the role of chronic illness. *Journal of Affective Disorders*.

[33] van der Slot W. M. A., Nieuwenhuijsen C., van den Berg-Emons R. J. G. (2012). Chronic pain, fatigue, and depressive symptoms in adults with spastic bilateral cerebral palsy. *Developmental Medicine and Child Neurology*.

[34] Hayter M. R., Dorstyn D. S. (2014). Resilience, self-esteem and self-compassion in adults with spina bifida. *Spinal Cord*.

[35] Garaigordobil M., Dura A., Pérez J. I. (2005). Psychopathological symptoms, behavioural problems, and self-concept/self-esteem: a study of adolescents aged 14 to 17 years old. *Annuary of Clinical and Healthy Psychology*.

[36] Alfeld-Liro C., Sigelman C. K. (1998). Sex differences in self-concept and symptoms of depression during the transition to college. *Journal of Youth and Adolescence*.

[37] Hoffmann J. P., Baldwin S. A., Cerbone F. G. (2003). Onset of major depressive disorder among adolescents. *Journal of the American Academy of Child and Adolescent Psychiatry*.

[38] Torres R., Fernández F. (1995). Self-esteem and value of health as determinants of adolescent health behavior. *The Journal of Adolescent Health*.

[39] Orth U., Robins R. W., Widaman K. F., Conger R. D. (2014). Is low self-esteem a risk factor for depression? Findings from a longitudinal study of mexican-origin youth. *Developmental Psychology*.

[40] Takakura M., Sakihara S. (2001). Psychosocial correlates of depressive symptoms among Japanese high school students. *The Journal of Adolescent Health*.

[41] Palacios E. G., Echaniz I. E., Fernández A. R., De Barrón I. C. O. (2015). Personal self-concept and satisfaction with life in adolescence, youth and adulthood. *Psicothema*.

[42] Lindwall M., Asci H., Crocker P. (2014). The physical self in motion: within-person change and associations of change in self-esteem, physical self-concept, and physical activity in adolescent girls. *Journal of Sport & Exercise Psychology*.

[43] ALGSA

[44] PatientCrossRoads.org https://connect.patientcrossroads.org/?org=algsa.

